# Pirfenidone and pentoxifylline attenuate acute cavernosal ischaemia–reperfusion injury in an experimental model of ischaemic priapism

**DOI:** 10.1186/s12894-026-02307-x

**Published:** 2026-08-11

**Authors:** İbrahim Göksoy, Arif Aydın, Fahriye Kılınç, İbrahim Kılınç, Mehmet Giray Sönmez

**Affiliations:** 1 Department of Urology, Health Sciences University Van Training and Research Hospital, Van, Türkiye; 2 Department of Urology, Necmettin Erbakan University, Konya, Türkiye; 3 Department of Pathology, Necmettin Erbakan University, Konya, Türkiye; 4 Department of Biochemistry, Necmettin Erbakan University, Konya, Türkiye

**Keywords:** Ischaemic priapism, Ischaemia–reperfusion injury, Pirfenidone, Pentoxifylline, Dexpanthenol, Cavernosal tissue

## Abstract

**Background:**

Ischaemic priapism is a urological emergency in which sustained cavernosal veno-occlusion produces progressive hypoxia, acidosis, smooth muscle compromise, and ultimately corporal fibrosis. Cavernosal injury is not confined to the ischaemic phase, as reperfusion following detumescence may further amplify tissue damage through oxidative stress, inflammatory activation, and endothelial dysfunction; adjunctive pharmacological strategies targeting this early reperfusion phase remain insufficiently characterised. This study was designed to evaluate and compare the acute effects of pirfenidone, pentoxifylline, dexpanthenol, and selected combination regimens on biochemical and histopathological markers of cavernosal injury in a rat model of ischaemic priapism-related ischaemia–reperfusion injury.

**Methods:**

Sixty-eight adult male Wistar albino rats were randomly allocated into seven groups: Sham, Control/IRI, pirfenidone, pentoxifylline, dexpanthenol, pirfenidone+dexpanthenol, and pentoxifylline+dexpanthenol. Ischaemic priapism was induced using a vacuum-assisted constriction-band model followed by a one-hour reperfusion period. Serum IL-1β, IL-10, malondialdehyde, and endothelial nitric oxide synthase levels were measured, and cavernosal tissues were evaluated by semi-quantitative histopathological scoring. Group comparisons used parametric or non-parametric tests with multiplicity adjustment. The study protocol was approved by the Animal Experiments Local Ethics Committee of Necmettin Erbakan University KONÜDAM Experimental Medicine Application and Research Center (22 December 2023; No. 2023-060). All procedures complied with institutional animal-care guidelines, and the study is reported in accordance with ARRIVE 2.0.

**Results:**

Sixty-seven animals were included in the final analysis. Relative to the Sham group, the Control/IRI group exhibited significantly elevated IL-1β, IL-10, malondialdehyde, and endothelial nitric oxide synthase levels alongside marked histopathological injury, confirming successful model induction. Pirfenidone significantly attenuated IL-1β and malondialdehyde levels and improved the composite pathology score. Pentoxifylline significantly reduced IL-1β, IL-10, malondialdehyde, and endothelial nitric oxide synthase levels and similarly improved the composite pathology score. Dexpanthenol attenuated malondialdehyde levels but did not consistently improve endothelial nitric oxide synthase or overall histopathological injury. Combination regimens produced endpoint-specific effects without consistently surpassing the corresponding monotherapies.

**Conclusions:**

Pirfenidone and pentoxifylline attenuated selected biochemical and histopathological components of acute cavernosal ischaemia–reperfusion injury in this experimental model. These findings are hypothesis-generating; additional studies incorporating long-term erectile function assessment, fibrotic remodelling markers, and pharmacokinetic evaluation are warranted to clarify the translational relevance.

**Supplementary Information:**

The online version contains supplementary material available at https://doi.org/10.1186/s12894-026-02307-x.

## Background

Priapism is an uncommon urological condition, with a reported incidence of approximately 5 cases per 100,000 men annually, and is classified by current guidelines into ischaemic (low-flow), non-ischaemic (high-flow), and recurrent ischaemic (stuttering) forms [1, 2]. Ischaemic priapism is a time-critical urological emergency, conventionally defined as a persistent penile erection exceeding four hours in duration and occurring independently of sexual stimulation. Sustained cavernosal veno-occlusion precipitates progressive hypoxia, acidosis, smooth muscle compromise, corporal fibrosis, and ultimately irreversible erectile dysfunction when intervention is delayed. Accordingly, emergency management is primarily directed toward prompt detumescence and restoration of cavernosal oxygenation [2, 3].

However, cavernosal injury in ischaemic priapism extends beyond the ischaemic phase itself. Reperfusion following detumescence may further amplify tissue damage through oxidative stress, inflammatory activation, endothelial dysfunction, and profibrotic signalling [4]. Even after technically successful detumescence, secondary reperfusion-related injury may continue to contribute to structural deterioration. Therefore, adjunctive pharmacological strategies targeting early reperfusion-related injury remain clinically relevant but insufficiently characterised [4, 5].

Pirfenidone is an antifibrotic agent with additional anti-inflammatory and antioxidant properties, mediated in part through modulation of transforming growth factor-β-related pathways [6]. Pentoxifylline, a methylxanthine derivative and non-selective phosphodiesterase inhibitor, has been shown to enhance microcirculatory perfusion and exert anti-inflammatory and antifibrotic effects in experimental models of cavernosal injury [7]. Dexpanthenol, a biologically active derivative of pantothenic acid, has demonstrated antioxidant and cytoprotective properties across several ischaemia–reperfusion models; however, its potential relevance to priapism-associated cavernosal injury has not been systematically evaluated [8, 9].

To capture complementary components of acute cavernosal ischaemia–reperfusion injury, IL-1β and MDA were selected as indicators of inflammatory activation and lipid peroxidation, respectively [10]. IL-10 was included to assess the counter-regulatory inflammatory response to ischaemia–reperfusion injury [11], whereas eNOS was evaluated as a marker of the endothelial nitric oxide pathway relevant to cavernosal physiology [2]. Collectively, these biomarkers were intended to assess inflammatory, oxidative, and endothelial components of injury and their modulation by the investigated treatments.

The present study was designed to evaluate and compare the acute effects of pirfenidone, pentoxifylline, dexpanthenol, and selected combination regimens on biochemical and histopathological markers of injury in a rat model of ischaemic priapism-related cavernosal ischaemia–reperfusion injury. We also examined whether combination therapy conferred incremental protective benefit beyond that achieved with monotherapy alone.

## Materials and methods

### Ethics statement and reporting guidelines

The study protocol was approved by the Animal Experiments Local Ethics Committee of Necmettin Erbakan University KONÜDAM Experimental Medicine Application and Research Center (decision date: 22 December 2023; decision no: 2023-060). All procedures complied with institutional guidelines for the care and use of laboratory animals and were conducted in accordance with the ARRIVE 2.0 reporting recommendations.

### Animals and housing conditions

A total of 68 adult male Wistar albino rats aged 8–10 weeks and weighing 175–245 g were enrolled. Animals were obtained from Necmettin Erbakan University KONÜDAM Experimental Medicine Application and Research Center, Konya, Türkiye. Animals were housed under controlled conditions with stable temperature and humidity, a 12-hour light/dark cycle, and ad libitum access to standard laboratory chow and water. All animals underwent a seven-day acclimatisation period prior to experimental procedures.

Only healthy adult male rats free of visible genital, systemic, or behavioural abnormalities were included. Animals that did not survive to protocol completion or in which successful induction of the priapism model could not be confirmed were excluded from the final analysis.

### Experimental design and group allocation

**Fig. 1:**
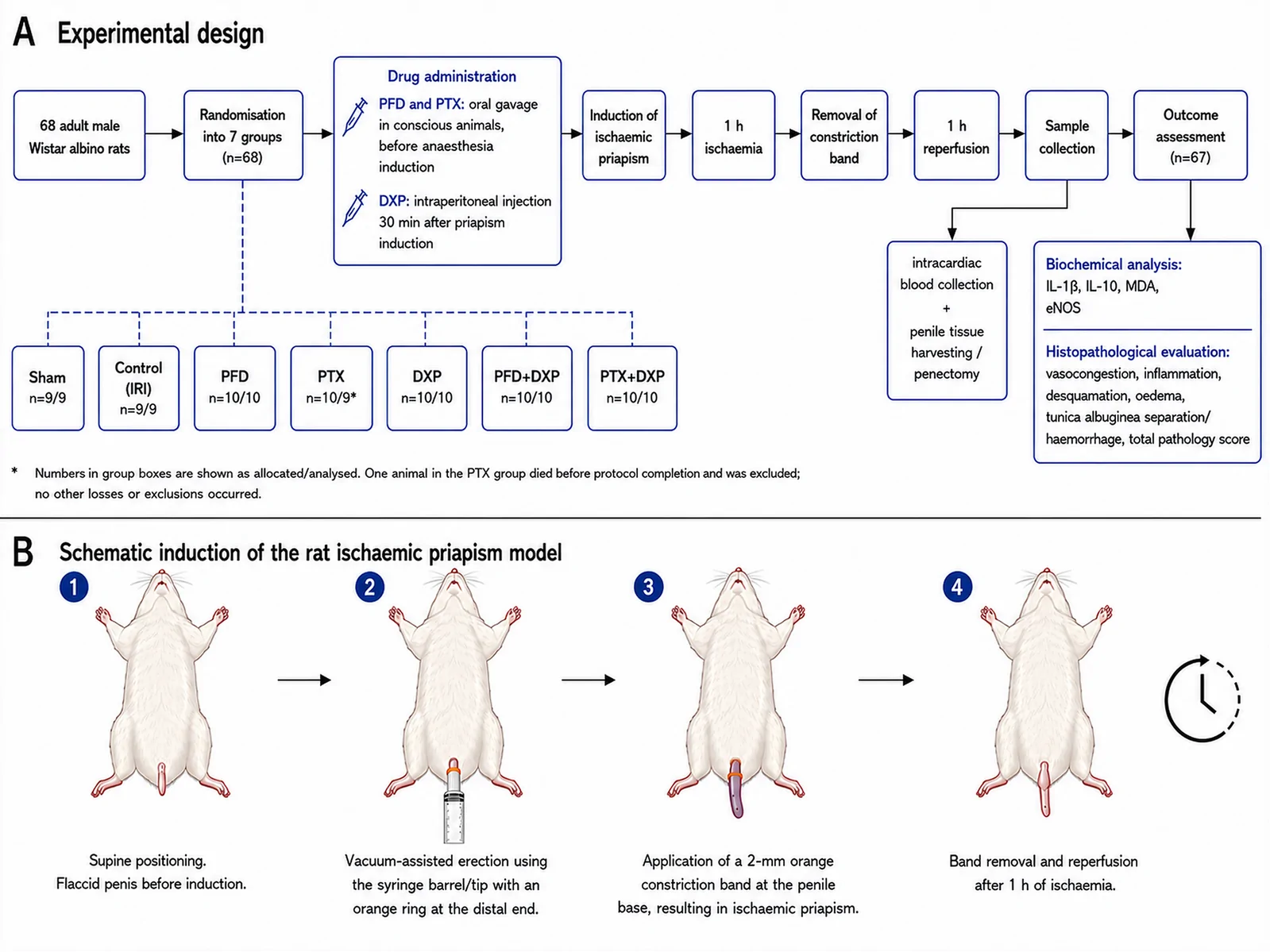
Schematic overview of the experimental design and induction of ischaemic priapism in the rat model. (**A**) Adult male Wistar albino rats were randomly allocated into seven experimental groups: sham, control, pirfenidone (PFD), pentoxifylline (PTX), dexpanthenol (DXP), pirfenidone + dexpanthenol (PFD + DXP), and pentoxifylline + dexpanthenol (PTX + DXP). PFD and PTX were administered via oral gavage to conscious animals before anaesthesia induction, while DXP was delivered intraperitoneally 30 min following priapism induction. Ischaemic priapism was sustained for one hour, after which the constriction band was removed and a subsequent one-hour reperfusion period was allowed to elapse. Penile tissue and blood samples were thereafter harvested for biochemical and histopathological evaluation. Numbers within the group boxes are presented as animals allocated/analysed. One animal in the PTX group died during the experimental procedure before outcome assessment; no other exclusions or losses occurred. (**B**) Sequential schematic illustration of the principal stages of the ischaemic priapism procedure. Following confirmation of adequate anaesthesia, each animal was placed in the supine position (1). A modified 50-mL syringe barrel was positioned over the penis to generate vacuum-assisted penile engorgement (2). A 2-mm constriction band fashioned from a 14-Fr Foley catheter segment was then advanced to the penile base to maintain veno-occlusion, and ischaemia was sustained for one hour (3). The constriction band was subsequently removed to permit reperfusion, which was allowed to proceed for one further hour before blood and tissue sampling (4). Panels are schematic and are not drawn to scale

This was an experimental, controlled animal study evaluating the acute effects of pirfenidone, pentoxifylline, dexpanthenol, and selected combination regimens in a rat model of ischaemic priapism-related cavernosal ischaemia–reperfusion injury. Animals were randomly allocated into seven groups: Sham, Control/IRI, PFD, PTX, DXP, PFD + DXP, and PTX + DXP. The experimental workflow is shown in Fig. 1A, and group allocation, interventions, doses, routes, and timing are summarised in Table 1.

**Table 1:** Experimental groups, intervention regimens, and timing of drug administration

Group	Experimental condition	*n* allocated / analysed	Intervention regimen	Route	Timing	Dose rationale
**Group 1**	Sham; no ischaemic priapism induction; penile tissue harvesting	9 / 9	Distilled water vehicle + physiological saline (0.9% sodium chloride) vehicle	Oral gavage + intraperitoneal injection	Oral vehicle before anaesthesia induction; intraperitoneal saline 30 min later	Vehicle administration matched to the oral and intraperitoneal treatment schedules
**Group 2**	Ischaemic priapism for 1 h followed by 1 h reperfusion	9 / 9	Distilled water vehicle + physiological saline (0.9% sodium chloride) vehicle	Oral gavage + intraperitoneal injection	Oral vehicle immediately before anaesthesia induction; intraperitoneal saline 30 min after priapism induction	Pharmacologically untreated IRI model with route- and timing-matched vehicle administration
**Group 3**	PFD	10 / 10	Pirfenidone, 325 mg/kg	Oral gavage	Immediately before anaesthesia induction	Based on previous experimental priapism protocols
**Group 4**	PTX	10 / 9	Pentoxifylline, single fixed dose of 100 mg	Oral gavage	Immediately before anaesthesia induction	Based on previous experimental priapism protocol
**Group 5**	DXP	10 / 10	Dexpanthenol, single fixed dose of 500 mg	Intraperitoneal injection	30 min after priapism induction	Based on previous experimental ischaemia–reperfusion and penile injury protocols
**Group 6**	PFD + DXP	10 / 10	Pirfenidone, 325 mg/kg + dexpanthenol, single fixed dose of 500 mg	Oral gavage + intraperitoneal injection	PFD immediately before anaesthesia induction; DXP 30 min after priapism induction	Combination regimen based on corresponding monotherapy protocols
**Group 7**	PTX + DXP	10 / 10	Pentoxifylline, single fixed dose of 100 mg + dexpanthenol, single fixed dose of 500 mg	Oral gavage + intraperitoneal injection	PTX immediately before anaesthesia induction; DXP 30 min after priapism induction	Combination regimen based on corresponding monotherapy protocols

*Abbreviations*: *DXP* dexpanthenol, *IRI* ischaemia–reperfusion injury, *PFD* pirfenidone, *PTX* pentoxifylline

One animal in the PTX group died before completion of the experimental protocol and was excluded from the final analysis. The Sham and Control/IRI groups received distilled water by oral gavage and physiological saline by intraperitoneal injection at time points corresponding to the treatment schedules. Pirfenidone was administered as a weight-adjusted dose, whereas pentoxifylline and dexpanthenol were administered as fixed single-dose regimens according to the referenced experimental protocols

### Randomisation and blinding

Animals were assigned to experimental groups by computer-generated simple randomisation prior to any intervention, and group allocation was finalised before initiation of experimental procedures.

Biochemical analyses were performed by a biochemistry specialist blinded to group allocation. Histopathological assessment and semi-quantitative scoring were conducted independently by an experienced pathologist who remained blinded to group identity throughout. Prior to analysis, all samples and datasets were coded to minimise assessment and interpretation bias.

### Drug preparation and administration

Pirfenidone was obtained as Pirfect^®^ 600 mg film-coated tablets (Nobel İlaç San. ve Tic. A.Ş., İstanbul, Türkiye), pentoxifylline as Trental^®^ CR 600 mg controlled-release film-coated tablets (Sanofi Sağlık Ürünleri Ltd. Şti., İstanbul, Türkiye), and dexpanthenol as Bepanthen^®^ 500 mg/2 mL solution for injection (Bayer Türk Kimya San. Ltd. Şti., İstanbul, Türkiye). Because commercially available finished medicinal products rather than analytical-grade active substances were used, numerical purity percentages were not applicable and the active-ingredient contents stated on the product labels were not independently assayed.

Immediately before administration, the pirfenidone and pentoxifylline tablets were finely pulverised and suspended in distilled water. For pirfenidone, the amount administered was individually adjusted according to body weight to provide a dose of 325 mg/kg [6], and each suspension was brought to a final volume of 1 mL per animal. Pentoxifylline was prepared as a suspension containing 100 mg/mL and administered as a fixed dose of 100 mg in a total volume of 1 mL per animal [7]. Both agents were administered by oral gavage to fully conscious animals before induction of anaesthesia; therefore, the swallowing reflex was preserved during administration.

Dexpanthenol was administered intraperitoneally 30 min after induction of ischaemic priapism directly from the commercial 500 mg/2 mL ampoule without further dilution, corresponding to a total volume of 2 mL per animal [12]. To control for the routes and volumes of administration, animals in the Sham and Control/IRI groups received 1 mL of distilled water by oral gavage at the time corresponding to oral drug administration and 2 mL of sterile 0.9% sodium chloride solution intraperitoneally at the time corresponding to dexpanthenol administration. The doses, routes, and timing of administration were based on previously published agent-specific experimental protocols rather than chosen to achieve pharmacokinetic equivalence between the investigated regimens [6, 7, 12].

### Anaesthesia

General anaesthesia was induced in all animals, including the Sham group, with intramuscular ketamine hydrochloride (50 mg/kg) and xylazine hydrochloride (5 mg/kg). Anaesthetic depth was continuously monitored by assessment of the pedal withdrawal reflex and respiratory pattern. When required, a supplemental ketamine dose equivalent to one-third of the initial dose (approximately 16.7 mg/kg) was administered to maintain adequate anaesthetic depth.

### Induction of the ischaemic priapism model and reperfusion injury

Ischaemic priapism was induced using the previously established vacuum-assisted constriction-band model described by Sanli et al. [13]. The procedure is illustrated in Fig. 1B. Following confirmation of adequate anaesthesia, each animal was placed in the supine position. A modified 50-mL syringe barrel was positioned over the penis to generate vacuum-assisted erection, after which a 2-mm constriction band fashioned from a 14-Fr Foley catheter segment was advanced to the penile base to maintain veno-occlusion.

The ischaemic period was sustained for one hour. The constriction band was then removed and reperfusion was permitted for one additional hour before blood and tissue sampling. In the sham group, penile tissue harvesting was performed without induction of priapism or reperfusion.

The one-hour ischaemia period followed by one hour of reperfusion was selected in accordance with previous acute rat models of priapism-related ischaemia–reperfusion injury using the same experimental window, to capture early reperfusion-associated biochemical and histopathological changes and evaluate short-term treatment effects [10, 14].

### Sample collection and euthanasia

Upon completion of the reperfusion period, intracardiac blood samples were obtained from all animals under deep anaesthesia, transferred into anticoagulant-free tubes, allowed to clot at room temperature for 15 min, and centrifuged at 4,000 rpm at 4 °C for 15 min; serum fractions were separated and stored at − 20 °C until biochemical analysis. Animals were subsequently euthanised by exsanguination, and penile tissue specimens were harvested immediately thereafter for histopathological evaluation.

### Biochemical analysis

Serum concentrations of inflammatory, oxidative stress, and endothelial-related markers were measured using commercially available enzyme-linked immunosorbent assay kits according to the manufacturers’ instructions.

Interleukin-1β (IL-1β; A.B.T.™, Cat. No. ABT1074Ra) and interleukin-10 (IL-10; A.B.T.™, Cat. No. ABT3187Ra) were measured to assess pro-inflammatory and counter-regulatory cytokine responses, respectively, and were expressed as pg/mL. Endothelial nitric oxide synthase (eNOS; A.B.T.™, Cat. No. ABT1421Ra) was measured as an endothelial nitric oxide pathway-related marker and expressed as pg/mL. Serum eNOS was included as an exploratory marker to enable parallel assessment alongside the other serum-based biochemical outcomes, while penile tissue was prioritised for histopathological evaluation. Malondialdehyde (MDA; A.B.T.™, Cat. No. ABT10165Ra) was measured as a marker of lipid peroxidation and expressed as ng/mL.

Total oxidant status (TOS; Elabscience, Cat. No. E-BC-K802-M) and total antioxidant status (TAS; Elabscience, Cat. No. E-BC-K801-M) were also measured as exploratory global indices of redox balance and expressed as mEquiv./L and mmol/L, respectively.

### Histopathological evaluation

Penile tissue specimens were fixed in 10% neutral buffered formalin, processed through an automated tissue processor, embedded in paraffin, and sectioned at approximately 4 μm. Sections were stained with haematoxylin–eosin for general morphological assessment and with Masson’s trichrome for evaluation of connective tissue changes.

All sections were examined under light microscopy by an experienced pathologist blinded to group allocation. Representative photomicrographs were acquired using an integrated digital imaging system.

Histopathological injury was graded semi-quantitatively using a scoring system adapted from previously published experimental models of priapism and cavernosal injury [10]. Five parameters were evaluated: vasocongestion, inflammation, desquamation, oedema, and tunica albuginea separation/haemorrhage. Each parameter was scored on a four-point ordinal scale (0 = absent or within normal limits; 1 = mild; 2 = moderate; 3 = severe), and a composite total pathology score was derived for each specimen by summing individual parameter scores, yielding a maximum possible score of 15.

As scoring was performed by a single blinded pathologist, formal inter-rater reliability could not be assessed; this represents an acknowledged limitation of the histopathological evaluation.

### Outcome measures

Primary outcomes were treatment-related differences in serum IL-1β, IL-10, malondialdehyde, and endothelial nitric oxide synthase levels and semi-quantitative histopathological injury scores. Total antioxidant status and total oxidant status were evaluated as exploratory global redox parameters.

### Statistical analysis

All analyses were performed using IBM SPSS Statistics for Windows, version 22.0 (IBM Corp., Armonk, NY, USA), with the individual animal as the unit of analysis. Continuous biochemical variables were summarised as mean ± standard deviation or median (minimum–maximum), according to their distribution, and ordinal histopathological scores as median (minimum–maximum). Normality was assessed using the Shapiro–Wilk test together with visual inspection of histograms and Q–Q plots, and homogeneity of variance was assessed using Levene’s test. IL-1β, IL-10, and MDA met the assumptions for parametric analysis, whereas eNOS did not and was analysed non-parametrically. Histopathological scores were analysed non-parametrically because of their ordinal nature.

A prespecified Sham-versus-Control/IRI contrast was performed first to confirm successful model induction, using an independent-samples t test or Mann–Whitney U test, as appropriate. This contrast addressed a separate model-induction question and was therefore not included in the multiplicity adjustments applied to the treatment comparisons.

Treatment effects were subsequently evaluated against the Control/IRI group. Parametrically analysed outcomes were compared using one-way analysis of variance, followed by Tukey’s honestly significant difference post-hoc test when the assumption of homogeneity of variance was met or the Games–Howell post-hoc test when variances were unequal. Non-normally distributed and ordinal outcomes were initially assessed using the Kruskal–Wallis test. Where the omnibus test was significant, five prespecified Mann–Whitney U comparisons were performed between the Control/IRI group and each treatment group, with Bonferroni adjustment applied separately within each outcome. Two-sided asymptotic p values without continuity correction were used for all Mann–Whitney U analyses.

Four additional, non-prespecified contrasts comparing each combination regimen with its component treatments were examined as exploratory secondary analyses: PFD + DXP versus PFD, PFD + DXP versus DXP, PTX + DXP versus PTX, and PTX + DXP versus DXP. For IL-1β, IL-10, and MDA, p values were obtained from the corresponding multiplicity-adjusted Tukey HSD or Games–Howell all-pairwise post-hoc analyses. For eNOS and the histopathological scores, the four Mann–Whitney U comparisons were treated as a separate exploratory family and Bonferroni-adjusted within each outcome. These results are reported in Additional file 1, Table S4.

No a priori sample-size calculation was performed because the study was exploratory and directly comparable preliminary data were unavailable for all evaluated regimens. Group sizes were based on previous experimental studies of priapism and ischaemia–reperfusion injury using comparable endpoints. A sensitivity analysis was conducted using G*Power version 3.1.9.7 (Heinrich-Heine-Universität Düsseldorf, Düsseldorf, Germany). Based on a one-way fixed-effects omnibus F test with 67 animals distributed across seven groups, α = 0.05, and 80% power, the minimum detectable effect size was Cohen’s f = 0.475. This value was considered an approximate omnibus reference and not a formal power estimate for the non-parametric outcomes.

Effect sizes for the five prespecified treatment-versus-Control/IRI comparisons were calculated using R version 4.6.1 (R Foundation for Statistical Computing, Vienna, Austria) and the effectsize package. Parametric comparisons were expressed as Hedges’ g and non-parametric comparisons as rank-biserial correlations. Corresponding 95% confidence intervals were estimated using the noncentrality-parameter method for Hedges’ g and Fisher’s transformation for rank-biserial correlations. All tests were two-tailed, and *p* < 0.05 was considered statistically significant.

## Results

### Animal flow and confirmation of model induction

A total of 68 adult male Wistar albino rats were randomly allocated into seven experimental groups. One animal in the PTX group died during the experimental procedure, before outcome assessment and was excluded from the final analysis; accordingly, 67 animals were included in the final statistical evaluation.

Successful induction of cavernosal ischaemia–reperfusion injury was confirmed by comparison of the Sham and Control/IRI groups. Relative to the Sham group, the Control/IRI group exhibited significantly elevated serum IL-1β, IL-10, MDA, and eNOS levels. Histopathological confirmation was further supported by significantly higher scores for vasocongestion, inflammation, desquamation, oedema, tunica albuginea separation/haemorrhage, and total pathology in the Control/IRI group.

Among the exploratory global redox parameters, TAS did not differ significantly between the Sham and Control/IRI groups, whereas TOS demonstrated a directionally discordant change relative to MDA. Both parameters were therefore considered to have limited interpretability as indices of oxidative injury within this acute model and are reported separately in Additional file 1.

### Biochemical findings

**Fig. 2:**
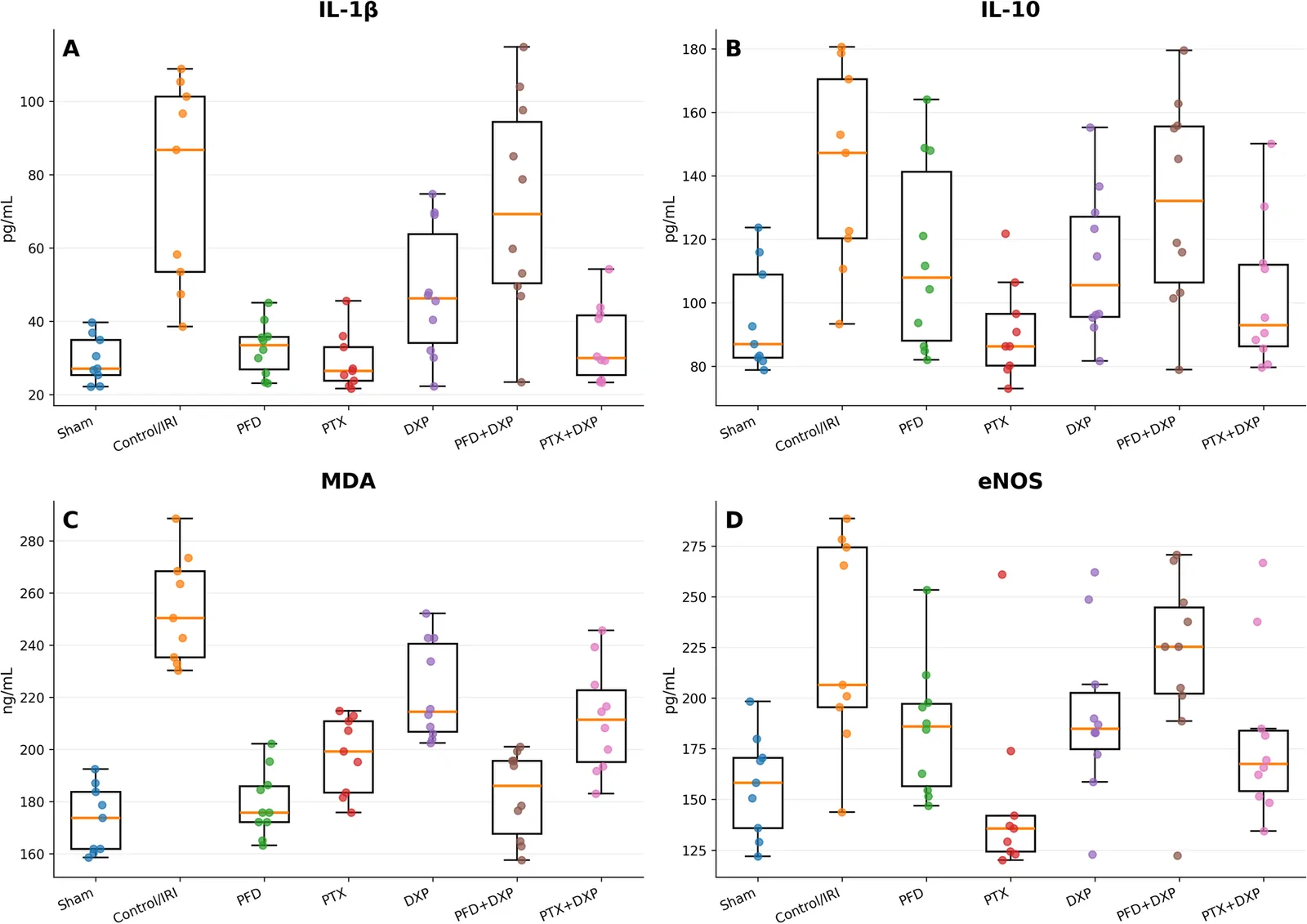
Distribution of biochemical outcomes across the experimental groups. Box plots show serum (**A**) interleukin-1β (IL-1β), (**B**) interleukin-10 (IL-10), (**C**) malondialdehyde (MDA), and (**D**) endothelial nitric oxide synthase (eNOS) levels. Boxes represent the median and interquartile range, whiskers extend to 1.5 times the interquartile range, and individual points represent measurements from individual animals. DXP, dexpanthenol; eNOS, endothelial nitric oxide synthase; IL, interleukin; IRI, ischaemia–reperfusion injury; MDA, malondialdehyde; PFD, pirfenidone; PTX, pentoxifylline

The main biochemical outcomes are summarised in Table 2 and graphically presented in Fig. 2.

**Table 2:** Main biochemical outcomes according to experimental groups

Biomarker	Sham	Control / IRI	PFD	PTX	DXP	PFD + DXP	PTX + DXP	Key comparisons vs. Control / IRI
**IL-1β**	29.56 ± 6.38	77.46 ± 27.72	32.65 ± 7.21	29.08 ± 7.79	47.91 ± 18.05	71.32 ± 29.31	34.12 ± 10.51	Sham, *p* < 0.001; PFD, *p* = 0.010; PTX, *p* = 0.006; PTX + DXP, *p* = 0.012
**IL-10**	94.98 ± 16.78	141.87 ± 31.58	114.48 ± 29.93	91.17 ± 15.17	112.02 ± 23.44	131.67 ± 32.46	102.38 ± 23.30	Sham, *p* = 0.002; PTX, *p* = 0.002; PTX + DXP, *p* = 0.026
**MDA**	173.11 ± 12.96	253.93 ± 20.55	179.24 ± 12.65	197.87 ± 14.72	222.15 ± 18.78	182.51 ± 16.57	211.71 ± 20.60	Sham, *p* < 0.001; PFD, *p* < 0.001; PTX, *p* < 0.001; DXP, *p* = 0.003; PFD + DXP, *p* < 0.001; PTX + DXP, *p* < 0.001
**eNOS**	158.30 (122.00–198.30)	206.50 (143.70–288.60)	186.05 (147.00–253.30)	135.80 (120.10–260.90)	185.00 (122.80–262.10)	225.40 (122.30–270.60)	167.55 (134.40–266.80)	Sham, *p* = 0.004; PTX, *p* = 0.012

Data for IL-1β, IL-10, and MDA are presented as mean ± standard deviation; eNOS is presented as median (minimum–maximum). The key comparisons column shows only statistically significant comparisons. IL-1β was analysed using Games–Howell and IL-10 and MDA using Tukey HSD post-hoc tests within the treatment-effect family comprising the Control/IRI and five treatment groups. For eNOS, Bonferroni correction was applied to the five prespecified Control/IRI-versus-treatment Mann–Whitney U comparisons. The Sham-versus-Control/IRI comparison was treated as a separate model-induction contrast. Detailed pairwise comparisons and effect sizes with 95% confidence intervals are provided in Additional file 1

*Abbreviations*: *DXP* dexpanthenol, *eNOS* endothelial nitric oxide synthase, *IL* interleukin, *IRI* ischaemia–reperfusion injury, *MDA* malondialdehyde, *PFD* pirfenidone, *PTX* pentoxifylline

Serum IL-1β levels were significantly elevated in the Control/IRI group relative to the Sham group. Among the treatment groups, IL-1β levels were significantly lower in the PFD, PTX, and PTX + DXP groups compared with the Control/IRI group. DXP monotherapy and the PFD + DXP combination did not yield a statistically significant reduction in IL-1β levels.

Serum IL-10 levels were likewise significantly elevated in the Control/IRI group relative to the Sham group. IL-10 levels were significantly lower in the PTX and PTX + DXP groups compared with the Control/IRI group; no statistically significant reduction was observed in the remaining treatment groups.

MDA levels were significantly higher in the Control/IRI group than in the Sham group. All treatment groups demonstrated significantly reduced MDA levels relative to the Control/IRI group.

Serum eNOS levels were significantly elevated in the Control/IRI group compared with the Sham group. Among the treatment groups, a significant reduction in eNOS levels was observed only in the PTX group relative to the Control/IRI group (*p* = 0.012); no significant differences were observed for the PFD, DXP, PFD + DXP, or PTX + DXP groups.

### Histopathological findings

**Fig. 3:**
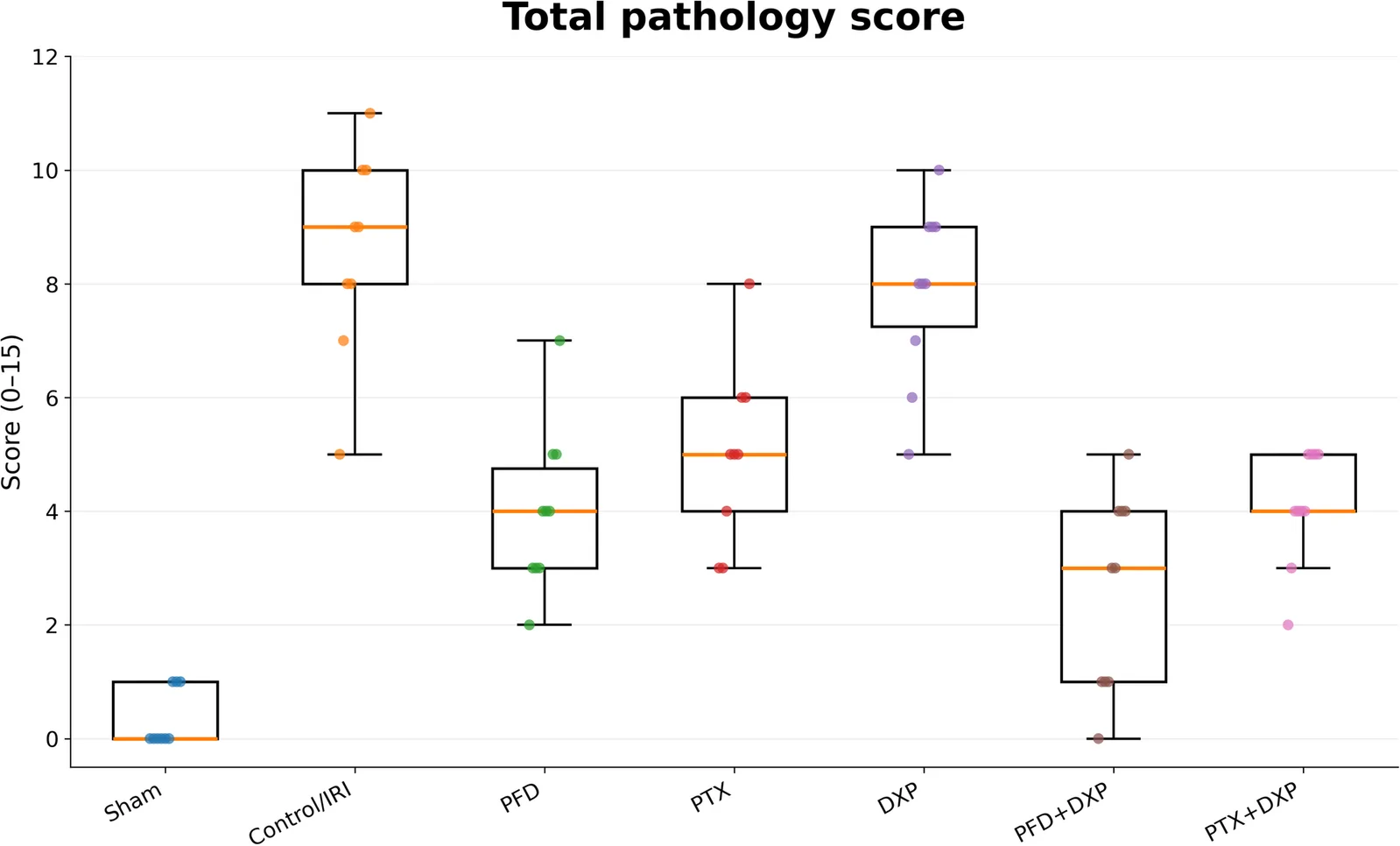
Distribution of the composite total pathology score across the experimental groups. Boxes represent the median and interquartile range, whiskers extend to 1.5 times the interquartile range, and individual points represent measurements from individual animals. The total pathology score was calculated by summing the five histopathological injury parameters—vasocongestion, inflammation, desquamation, oedema, and tunica albuginea separation/haemorrhage—and ranged from 0 to 15. DXP, dexpanthenol; IRI, ischaemia–reperfusion injury; PFD, pirfenidone; PTX, pentoxifylline

**Fig. 4:**
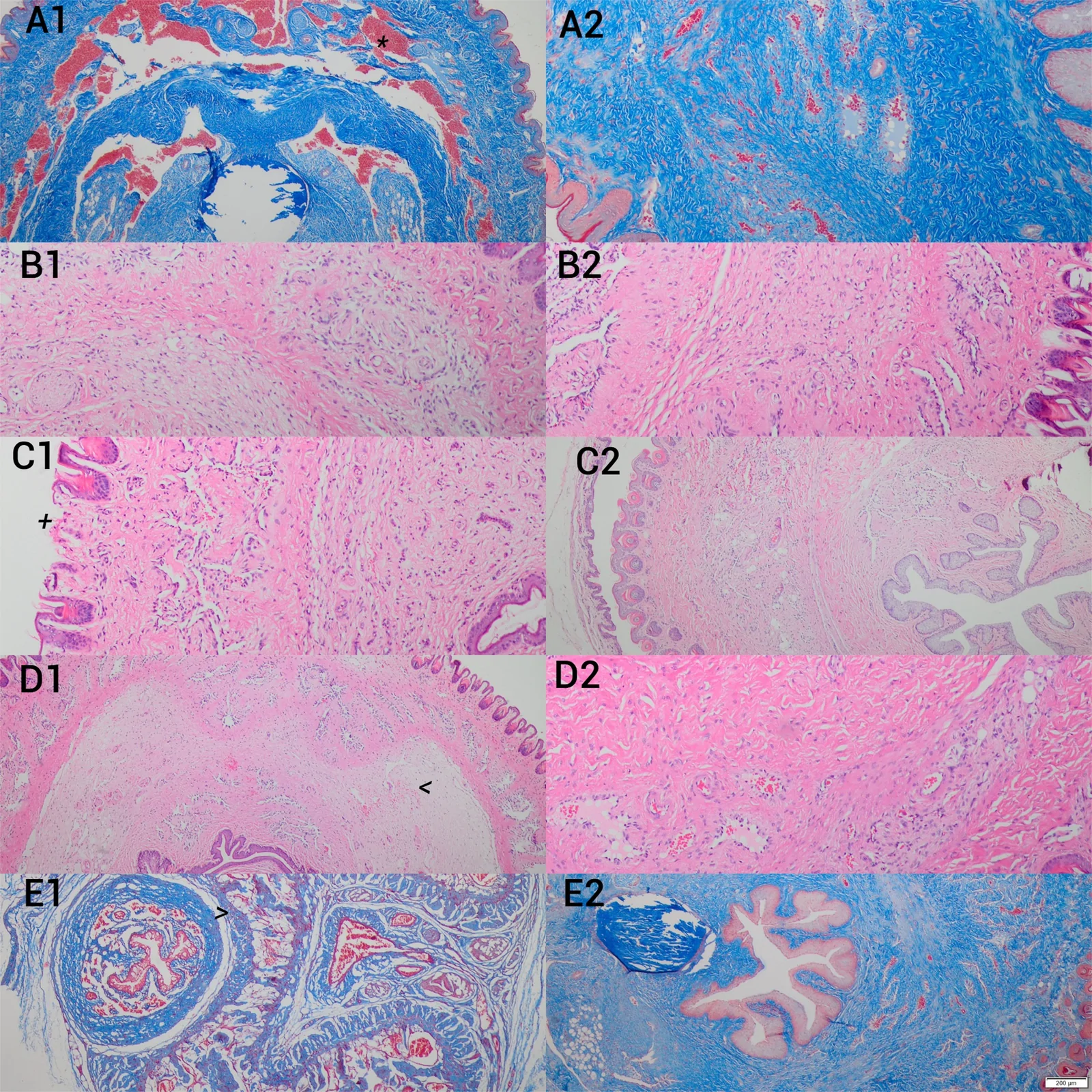
Representative histopathological findings in penile tissue following ischaemic priapism-related cavernosal ischaemia–reperfusion injury. (A1) Marked cavernosal vasocongestion in the Control/IRI group; Masson’s trichrome, ×40. (A2) Representative cavernosal vasocongestion in the PTX group; Masson’s trichrome, ×100. (B1) Prominent inflammatory cell infiltration in the DXP group; H&E, ×100. (B2) Attenuated inflammatory cell infiltration in the PFD group; H&E, ×100. (C1) Epithelial desquamation in the DXP group; H&E, ×40. (C2) Preserved epithelial integrity in the PTX + DXP group; H&E, ×40. (D1) Marked interstitial oedema in the Control/IRI group; H&E, ×100. (D2) Attenuated interstitial oedema in the PFD + DXP group; H&E, ×100. (E1) Tunica albuginea separation with haemorrhage in the DXP group; Masson’s trichrome, ×40. (E2) Preserved tunica albuginea architecture in the PFD group; Masson’s trichrome, ×40. Scale bar = 200 μm (E2). Annotations: asterisk (*), area of cavernosal vasocongestion; plus sign (+), area of epithelial desquamation; less-than sign (< ), area of interstitial oedema; greater-than sign (> ), area of tunica albuginea separation/haemorrhage. Original magnifications are indicated for each panel

Histopathological outcomes are summarised in Table 3, with the total pathology score graphically presented in Fig. 3 and representative photomicrographs shown in Fig. 4.

**Table 3:** Histopathological injury scores according to experimental groups

Histopathological parameter	Sham	Control / IRI	PFD	PTX	DXP	PFD + DXP	PTX + DXP	Key comparisons vs. Control / IRI
**Vasocongestion**	0 (0–1)	2 (1–3)	1 (1–3)	2 (1–3)	1 (1–2)	1 (0–2)	1 (1–2)	Sham, *p* < 0.001; PFD + DXP, *p* = 0.045
**Inflammation**	0 (0–0)	2 (1–2)	1 (0–1)	1 (1–1)	1 (0–2)	1 (0–1)	0 (0–1)	Sham, *p* < 0.001; PFD, *p* = 0.015; PFD + DXP, *p* = 0.015; PTX + DXP, *p* < 0.001
**Desquamation**	0 (0–0)	1 (1–2)	0 (0–1)	0 (0–1)	1 (0–2)	0 (0–1)	0 (0–1)	Sham, *p* < 0.001; PFD, *p* < 0.001; PTX, *p* = 0.004; PFD + DXP, *p* < 0.001; PTX + DXP, *p* < 0.001
**Oedema**	0 (0–0)	2 (2–3)	1.5 (1–2)	1 (0–3)	2 (1–2)	1 (0–1)	1 (1–2)	Sham, *p* < 0.001; PFD, *p* = 0.043; PFD + DXP, *p* < 0.001; PTX + DXP, *p* = 0.020
**Tunica albuginea separation/haemorrhage**	0 (0–0)	1 (1–2)	0.5 (0–1)	1 (0–1)	2 (0–2)	0 (0–1)	1 (0–2)	Sham, *p* < 0.001; PFD, *p* = 0.043; PFD + DXP, *p* = 0.008
**Total pathology score**	0 (0–1)	9 (5–11)	4 (2–7)	5 (3–8)	8 (5–10)	3 (0–5)	4 (2–5)	Sham, *p* < 0.001; PFD, *p* = 0.002; PTX, *p* = 0.012; PFD + DXP, *p* = 0.001; PTX + DXP, *p* = 0.002

Data are presented as median (minimum–maximum). Histopathological injury was graded semi-quantitatively from 0 to 3 for each parameter: 0 = absent or within normal limits, 1 = mild, 2 = moderate, and 3 = severe. The total pathology score was calculated by summing the five individual injury scores, yielding a possible range of 0–15. For each outcome, Bonferroni correction was applied to the five prespecified Control/IRI-versus-treatment Mann–Whitney U comparisons; the Sham-versus-Control/IRI comparison was treated as a separate model-induction contrast. The key comparisons column shows only statistically significant comparisons. Detailed pairwise comparisons and effect sizes with 95% confidence intervals are provided in Additional file 1. A two-tailed p value < 0.050 was considered statistically significant

*Abbreviations*: *DXP* dexpanthenol, *IRI* ischaemia–reperfusion injury, *PFD* pirfenidone, *PTX* pentoxifylline

The Control/IRI group exhibited marked cavernosal tissue injury relative to the Sham group, characterised by increased vasocongestion, inflammatory cell infiltration, desquamation, oedema, tunica albuginea separation/haemorrhage, and a higher composite total pathology score.

Vasocongestion showed limited treatment responsiveness; only the PFD + DXP group had significantly lower scores than the Control/IRI group (*p* = 0.045), whereas no significant differences were observed for the other treatment groups.

Inflammation scores were significantly lower in the PFD (*p* = 0.015), PFD + DXP (*p* = 0.015), and PTX + DXP (*p* < 0.001) groups relative to the Control/IRI group. The differences observed for PTX and DXP monotherapy did not reach statistical significance.

Desquamation scores were significantly lower in the PFD (*p* < 0.001), PTX (*p* = 0.004), PFD + DXP (*p* < 0.001), and PTX + DXP (*p* < 0.001) groups compared with the Control/IRI group.

Oedema scores were significantly lower in the PFD (*p* = 0.043), PFD + DXP (*p* < 0.001), and PTX + DXP (*p* = 0.020) groups relative to the Control/IRI group; the differences observed for PTX and DXP monotherapy were not statistically significant.

Tunica albuginea separation/haemorrhage scores were significantly lower in the PFD (*p* = 0.043) and PFD + DXP (*p* = 0.008) groups relative to the Control/IRI group; no statistically significant differences were observed for PTX, DXP, or PTX + DXP.

The composite total pathology score was significantly higher in the Control/IRI group than in the Sham group, confirming successful model induction. Relative to the Control/IRI group, total pathology scores remained significantly lower in the PFD (*p* = 0.002), PTX (*p* = 0.012), PFD + DXP (*p* = 0.001), and PTX + DXP (*p* = 0.002) groups, whereas DXP monotherapy did not achieve a statistically significant reduction.

Overall, pirfenidone and pentoxifylline demonstrated the most consistent attenuation across evaluated endpoints. Pirfenidone reduced IL-1β and malondialdehyde levels and improved the composite pathology score, whereas pentoxifylline reduced IL-1β, IL-10, malondialdehyde, and endothelial nitric oxide synthase levels and similarly improved the composite pathology score. Dexpanthenol attenuated malondialdehyde levels only, and in exploratory direct contrasts the combination regimens differed from dexpanthenol monotherapy across several histopathological endpoints but from the corresponding pirfenidone or pentoxifylline monotherapies only at individual endpoints (Additional file 1, Table S4).

## Discussion

The present study investigated the acute effects of pirfenidone, pentoxifylline, dexpanthenol, and selected combination regimens on cavernosal ischaemia–reperfusion injury in an experimental rat model of ischaemic priapism. The principal finding was that pirfenidone and pentoxifylline attenuated multiple biochemical and histopathological components of acute cavernosal injury, whereas dexpanthenol exerted a more limited effect confined principally to lipid peroxidation. Combination regimens produced endpoint-specific effects without consistently surpassing the corresponding monotherapies.

Ischaemic priapism is characterised by progressive cavernosal hypoxia, acidosis, impaired perfusion, smooth muscle injury, and, when intervention is delayed, irreversible fibrosis and erectile dysfunction [2, 15, 16]. Beyond the primary ischaemic insult, reperfusion following detumescence may paradoxically exacerbate tissue damage through oxidative stress, endothelial dysfunction, inflammatory activation, and profibrotic signalling [4, 17]. In the present study, the untreated Control/IRI group exhibited significantly elevated IL-1β, MDA, IL-10 and eNOS levels alongside prominent histopathological injury, collectively indicating successful reproduction of an acute priapism-related cavernosal injury phenotype. These findings support the pathophysiological relevance of inflammatory, oxidative, and endothelial-related mechanisms during the early phase of cavernosal ischaemia–reperfusion injury. Because a previously validated model of ischaemic priapism was employed, the present findings should be interpreted as a pharmacological comparison within an established model rather than as a validation of the model itself.

The cytokine profile observed in the present study warrants cautious interpretation. The elevation of IL-1β in the Control/IRI group was consistent with acute pro-inflammatory activation following cavernosal ischaemia–reperfusion injury. IL-10 was likewise elevated in the Control/IRI group relative to the Sham group. Although IL-10 is conventionally regarded as an anti-inflammatory mediator, its increase in the untreated injury group should not be interpreted as evidence of effective inflammatory resolution. Rather, this elevation may reflect a counter-regulatory response to acute tissue injury, as context-dependent IL-10 induction has been described in other experimental ischaemia–reperfusion settings. Accordingly, the lower IL-10 levels observed in PTX-containing regimens should be interpreted in conjunction with the broader pattern of reduced IL-1β and MDA levels and, for PTX monotherapy, the reduction in eNOS, rather than as isolated suppression of an anti-inflammatory pathway [11, 18].

The elevation of eNOS in the Control/IRI group may reflect an acute endothelial response involving nitric oxide pathway activation during cavernosal ischaemia–reperfusion injury. Although nitric oxide signalling is integral to cavernosal vascular regulation, dysregulated nitric oxide-related activity may coexist with oxidative and inflammatory injury during reperfusion. A significant reduction in serum eNOS was observed only with pentoxifylline monotherapy, whereas no significant change was detected with the other treatment regimens. This pattern suggests that the effects of the other agents, including pirfenidone, may be more closely related to inflammatory and oxidative pathways than to changes in circulating eNOS. Accordingly, circulating eNOS was interpreted as an exploratory, indirect indicator of nitric oxide pathway perturbation rather than as a surrogate for tissue-level eNOS expression or cavernosal endothelial function.

Pirfenidone attenuated several injury-related endpoints in this acute model, with significant reductions in IL-1β and MDA levels and improvement in both the composite pathology score and selected histopathological parameters. This finding is biologically plausible given that pirfenidone possesses established antifibrotic, anti-inflammatory, and antioxidant properties, mediated in part through modulation of transforming growth factor-β-related signalling [19]. Previous experimental work in ischaemic priapism has indicated that pirfenidone may improve erectile function and attenuate fibrosis-related cavernosal injury [6]. The present findings extend this rationale by demonstrating that pirfenidone may also reduce selected early inflammatory, oxidative, and structural changes during the acute ischaemia–reperfusion phase. Nevertheless, as erectile function and long-term fibrotic remodelling were not assessed in the present study, these results should be interpreted as evidence of early tissue-level attenuation of injury rather than direct proof of preserved erectile function.

Pentoxifylline also attenuated several biochemical and histopathological endpoints in the acute setting. PTX significantly reduced IL-1β, IL-10, MDA, and eNOS levels relative to the Control/IRI group and improved the composite pathology score. As a methylxanthine derivative with rheological, anti-inflammatory, and antifibrotic properties, pentoxifylline has previously been investigated in experimental priapism, cavernosal tissue injury, and fibrosis-related contexts [7, 20, 21]. The present findings support its potential relevance during the acute phase of priapism-related cavernosal injury. As noted above, the reduction in IL-10 in PTX-containing regimens should not be interpreted in isolation as adverse suppression of anti-inflammatory activity. When considered together with the parallel reductions in IL-1β, MDA, eNOS, and histopathological injury, it more plausibly reflects attenuation of the broader injury-associated inflammatory response.

Dexpanthenol exerted a narrower effect than pirfenidone or pentoxifylline. Although DXP reduced MDA, this did not translate into consistent improvement in eNOS-related parameters or composite histopathological injury. Prior studies have reported antioxidant and tissue-repair effects of dexpanthenol in renal, testicular, and penile injury models [8, 9, 12, 22]. The limited effect observed here may reflect tissue-specific differences, the severity of the acute cavernosal insult, the short reperfusion window, or the post-induction intraperitoneal schedule. Taken together, the broader pattern of effects observed with pentoxifylline may reflect its combined rheological, anti-inflammatory, and antioxidant actions, whereas pirfenidone showed a profile more closely aligned with inflammatory and oxidative endpoints, and dexpanthenol produced a predominantly lipid peroxidation-related response.

Combination regimens produced endpoint-specific effects without consistent additive benefit over the corresponding monotherapies. Relative to the Control/IRI group, both combinations attenuated malondialdehyde levels and improved the composite pathology score, and both differed from dexpanthenol monotherapy across several histopathological endpoints; compared with the corresponding pirfenidone or pentoxifylline monotherapies, however, differences were limited to isolated endpoints—higher IL-1β (*p* = 0.021) and lower oedema scores (*p* = 0.020) with PFD + DXP than with pirfenidone alone, and lower inflammation scores with PTX + DXP than with pentoxifylline alone (*p* < 0.001) (Additional file 1, Table S4). Because these contrasts were not prespecified and were examined across ten outcomes, the isolated differences are regarded as hypothesis-generating rather than as evidence of additive efficacy. These findings suggest that combining agents with antioxidant or anti-inflammatory properties does not necessarily confer superior acute cavernosal tissue protection. A PFD + PTX combination arm was not included; therefore, potential additive or synergistic effects between these two agents could not be evaluated and should be investigated in future studies.

The oxidative stress findings also warrant cautious interpretation. MDA demonstrated a clear and consistent increase in the untreated Control/IRI group and was significantly reduced across all treatment groups, supporting its utility as the principal lipid peroxidation marker in this model. In contrast, TAS did not differentiate between the Sham and Control/IRI groups, while TOS showed a directionally discordant change relative to MDA. This discrepancy probably reflects the fact that MDA is a cumulative end-product of lipid peroxidation, whereas TAS and TOS represent instantaneous measures of oxidant and antioxidant pools that are subject to rapid consumption and replenishment; within a short acute reperfusion window a compensatory antioxidant response may therefore mask the net oxidant burden. Limited sensitivity of these global colorimetric indices may contribute additionally. Oxidative injury was therefore interpreted primarily through MDA, with TAS and TOS regarded as exploratory global redox parameters. Accordingly, OSI was not calculated because a ratio derived from these discordant parameters was unlikely to provide a robust additional measure of oxidative injury.

From a translational perspective, the present findings support further investigation of pirfenidone and pentoxifylline as candidate adjunctive agents for limiting early cavernosal tissue injury following ischaemic priapism. Nevertheless, these results should be regarded as hypothesis-generating rather than practice-changing. Current emergency management of ischaemic priapism remains centred on prompt detumescence and restoration of cavernosal oxygenation [3]. Adjunctive pharmacological strategies targeting secondary inflammatory, oxidative, and fibrotic injury require validation in models incorporating functional erectile endpoints, extended reperfusion periods, and chronic fibrosis assessment before clinically meaningful conclusions can be drawn [6, 7].

Several limitations of the present study warrant acknowledgement. First, the one-hour ischaemia period followed by one hour of reperfusion constituted a short, acute experimental window designed to assess early injury and single-dose treatment effects. This design does not reproduce prolonged clinical priapism or later tissue remodelling and precludes conclusions regarding long-term fibrosis, erectile function, or the durability of treatment effects. Second, functional erectile parameters—particularly intracavernosal pressure responses—were not measured. Third, molecular analyses including TGF-β1, collagen I/III, α-SMA, NF-κB signalling, apoptotic markers, and immunohistochemical evaluation were not performed, which limits mechanistic interpretation of the observed effects. Fourth, the regimens differed in dose expression, route, and timing, and plasma or tissue drug concentrations were not measured; therefore, differences in systemic exposure cannot be excluded and pharmacokinetic equivalence across agents cannot be assumed. Fifth, the pre-induction administration of pirfenidone and pentoxifylline, in contrast to the post-induction administration of dexpanthenol, primarily represents a preventive proof-of-concept approach and does not directly reproduce clinical treatment after priapism onset; future studies should therefore evaluate delayed administration during established ischaemia or reperfusion. Sixth, although all assays were performed in a single batch per analyte with concentrations falling within the manufacturers’ specified detection ranges, the individual absorbance values of the ELISA standard wells were not retained in the archived analytical records, which precluded retrospective reporting of calibration performance. Finally, no formal a priori sample-size calculation was performed; although the sensitivity analysis characterised the detectable effect magnitude with the final sample, smaller treatment effects may have remained undetected, and the findings should therefore be interpreted as exploratory.

## Conclusions

Pirfenidone and pentoxifylline attenuated selected biochemical and histopathological components of acute cavernosal ischaemia–reperfusion injury in this experimental model of ischaemic priapism. Dexpanthenol exerted a more limited effect, principally reflected by attenuation of lipid peroxidation without consistent structural or eNOS-related improvement. Combination regimens did not confer consistent additive benefit over the corresponding monotherapies. Further studies incorporating long-term erectile function assessment, fibrotic remodelling markers, and pharmacokinetic evaluation are warranted to clarify the translational relevance of these findings.

## Supplementary Information

Supplementary Material 1: Additional file 1 (.docx). Supplementary biochemical and histopathological outcomes. Contains Tables S1–S4 reporting exploratory TAS and TOS outcomes, detailed biochemical and histopathological comparisons with effect sizes and 95% confidence intervals, and exploratory combination-versus-component comparisons.

## Data Availability

The data supporting the findings of this study are available from the corresponding author upon reasonable request.

## Abbreviations

α-SMA: Alpha-smooth muscle actin
ARRIVE: Animal Research: Reporting of In Vivo Experiments
DXP: Dexpanthenol
ELISA: Enzyme-linked immunosorbent assay
eNOS: Endothelial nitric oxide synthase
H&E: Haematoxylin and eosin
IL-1β: Interleukin-1 beta
IL-10: Interleukin-10
IRI: Ischaemia–reperfusion injury
MDA: Malondialdehyde
NF-κB: Nuclear factor kappa B
OSI: Oxidative stress index
PFD: Pirfenidone
PTX: Pentoxifylline
TAS: Total antioxidant status
TGF-β1: Transforming growth factor-beta 1
TOS: Total oxidant status
