# Supplementary material for: Pirfenidone and pentoxifylline attenuate acute cavernosal ischaemia–reperfusion injury in an experimental model of ischaemic priapism
Source: BMC Urol. 2026 Aug 11;26:282. doi: 10.1186/s12894-026-02307-x (PMC13746569; doi:10.1186/s12894-026-02307-x)
Supplement: Supplementary file 1 [file 12894_2026_2307_MOESM1_ESM.pdf]

Additional file 1

Supplementary biochemical and histopathological outcomes

For: Pirfenidone and Pentoxifylline Attenuate Acute Cavernosal Ischaemia-Reperfusion Injury in an Experimental Model of Ischaemic Priapism

Supplementary Table S1. Exploratory TAS and TOS outcomes according to experimental groups

Parameter	Sham	Control/IRI	PFD	PTX	DXP	PFD+DXP	PTX+DXP	Model-induction contrast
**TAS**	0.324 ± 0.062	0.430 ± 0.165	0.333 ± 0.071	0.328 ± 0.072	0.513 ± 0.310	0.485 ± 0.102	0.391 ± 0.077	p=0.091
**TOS**	24.078 ± 2.593	20.378 ± 2.108	20.110 ± 2.561	22.678 ± 3.253	19.210 ± 3.684	19.380 ± 3.636	22.120 ± 2.527	p=0.004; direction not concordant with MDA

Data are presented as mean ± standard deviation. TAS and TOS were retained as exploratory global redox indices and were not used for the main treatment-effect interpretation.
Abbreviations: DXP, dexpanthenol; IRI, ischaemia–reperfusion injury; MDA, malondialdehyde; PFD, pirfenidone; PTX, pentoxifylline; TAS, total antioxidant status; TOS, total oxidant status.

Supplementary Table S2. Detailed biochemical comparisons versus the Control/IRI group, with effect sizes and 95% confidence intervals

Biomarker	Comparison	p value	Effect-size metric	Estimate	95% CI
**IL-1β**	Sham vs Control/IRI	p<0.001	—	—	—
**IL-1β**	PFD vs Control/IRI	p=0.010	Hedges' g	-2.169	[-3.274, -1.027]
**IL-1β**	PTX vs Control/IRI	p=0.006	Hedges' g	-2.262	[-3.416, -1.069]
**IL-1β**	DXP vs Control/IRI	p=0.134	Hedges' g	-1.221	[-2.158, -0.255]
**IL-1β**	PFD+DXP vs Control/IRI	p=0.997	Hedges' g	-0.205	[-1.065, 0.661]
**IL-1β**	PTX+DXP vs Control/IRI	p=0.012	Hedges' g	-2.019	[-3.093, -0.909]
**IL-10**	Sham vs Control/IRI	p=0.002	—	—	—
**IL-10**	PFD vs Control/IRI	p=0.243	Hedges' g	-0.851	[-1.746, 0.066]
**IL-10**	PTX vs Control/IRI	p=0.002	Hedges' g	-1.949	[-3.035, -0.824]
**IL-10**	DXP vs Control/IRI	p=0.165	Hedges' g	-1.034	[-1.948, -0.094]
**IL-10**	PFD+DXP vs Control/IRI	p=0.961	Hedges' g	-0.304	[-1.166, 0.567]
**IL-10**	PTX+DXP vs Control/IRI	p=0.026	Hedges' g	-1.371	[-2.330, -0.381]
**MDA**	Sham vs Control/IRI	p<0.001	—	—	—
**MDA**	PFD vs Control/IRI	p<0.001	Hedges' g	-4.238	[-5.874, -2.567]
**MDA**	PTX vs Control/IRI	p<0.001	Hedges' g	-2.987	[-4.319, -1.616]
**MDA**	DXP vs Control/IRI	p=0.003	Hedges' g	-1.546	[-2.533, -0.527]
**MDA**	PFD+DXP vs Control/IRI	p<0.001	Hedges' g	-3.678	[-5.157, -2.162]
**MDA**	PTX+DXP vs Control/IRI	p<0.001	Hedges' g	-1.960	[-3.022, -0.862]
**eNOS**	Sham vs Control/IRI	p=0.004	—	—	—
**eNOS**	PFD vs Control/IRI	p=0.395	Rank-biserial r	-0.478	[-0.783, 0.013]
**eNOS**	PTX vs Control/IRI	p=0.012	Rank-biserial r	-0.852	[-0.948, -0.614]
**eNOS**	DXP vs Control/IRI	p=0.512	Rank-biserial r	-0.444	[-0.766, 0.056]
**eNOS**	PFD+DXP vs Control/IRI	p=1.000	Rank-biserial r	-0.067	[-0.537, 0.436]
**eNOS**	PTX+DXP vs Control/IRI	p=0.169	Rank-biserial r	-0.578	[-0.831, -0.125]

For IL-1β, Games–Howell post-hoc p values are reported; for IL-10 and MDA, Tukey HSD post-hoc p values are reported. These procedures control the family-wise error rate across the six groups included in the treatment-effect analysis (Control/IRI and five treatment groups). For eNOS, the five prespecified Control/IRI-versus-treatment Mann–Whitney U comparisons were Bonferroni-adjusted within the outcome (adjusted p=min[5×raw p, 1]). The Sham-versus-Control/IRI comparison was analysed separately as a model-induction contrast and was not included in the treatment-comparison family.

Hedges' g was estimated using the pooled standard deviation and exact small-sample correction. Rank-biserial correlations were estimated from average ranks. Negative effect sizes indicate lower values in the treatment group than in the Control/IRI group. The 95% confidence intervals are unadjusted intervals for the effect-size estimates; multiplicity adjustment applies to the reported treatment-comparison p values.

Abbreviations: CI, confidence interval; DXP, dexpanthenol; eNOS, endothelial nitric oxide synthase; IL, interleukin; IRI, ischaemia–reperfusion injury; MDA, malondialdehyde; PFD, pirfenidone; PTX, pentoxifylline.

Supplementary Table S3. Detailed histopathological comparisons versus the Control/IRI group, with effect sizes and 95% confidence intervals

Histopathological parameter	Comparison	p value	Effect-size metric	Estimate	95% CI
**Vasocongestion**	Sham vs Control/IRI	p<0.001	—	—	—
**Vasocongestion**	PFD vs Control/IRI	p=0.391	Rank-biserial r	-0.422	[-0.755, 0.083]
**Vasocongestion**	PTX vs Control/IRI	p=1.000	Rank-biserial r	0.000	[-0.499, 0.499]
**Vasocongestion**	DXP vs Control/IRI	p=0.991	Rank-biserial r	-0.311	[-0.694, 0.209]
**Vasocongestion**	PFD+DXP vs Control/IRI	p=0.045	Rank-biserial r	-0.644	[-0.862, -0.228]
**Vasocongestion**	PTX+DXP vs Control/IRI	p=0.991	Rank-biserial r	-0.311	[-0.694, 0.209]
**Inflammation**	Sham vs Control/IRI	p<0.001	—	—	—
**Inflammation**	PFD vs Control/IRI	p=0.015	Rank-biserial r	-0.733	[-0.899, -0.382]
**Inflammation**	PTX vs Control/IRI	p=0.053	Rank-biserial r	-0.556	[-0.826, -0.078]
**Inflammation**	DXP vs Control/IRI	p=0.116	Rank-biserial r	-0.544	[-0.816, -0.077]
**Inflammation**	PFD+DXP vs Control/IRI	p=0.015	Rank-biserial r	-0.733	[-0.899, -0.382]
**Inflammation**	PTX+DXP vs Control/IRI	p<0.001	Rank-biserial r	-0.956	[-0.984, -0.876]
**Desquamation**	Sham vs Control/IRI	p<0.001	—	—	—
**Desquamation**	PFD vs Control/IRI	p<0.001	Rank-biserial r	-0.944	[-0.981, -0.847]
**Desquamation**	PTX vs Control/IRI	p=0.004	Rank-biserial r	-0.877	[-0.957, -0.671]
**Desquamation**	DXP vs Control/IRI	p=0.356	Rank-biserial r	-0.400	[-0.743, 0.109]
**Desquamation**	PFD+DXP vs Control/IRI	p<0.001	Rank-biserial r	-0.944	[-0.981, -0.847]
**Desquamation**	PTX+DXP vs Control/IRI	p<0.001	Rank-biserial r	-0.944	[-0.981, -0.847]
**Oedema**	Sham vs Control/IRI	p<0.001	—	—	—
**Oedema**	PFD vs Control/IRI	p=0.043	Rank-biserial r	-0.611	[-0.847, -0.175]
**Oedema**	PTX vs Control/IRI	p=0.073	Rank-biserial r	-0.630	[-0.859, -0.190]
**Oedema**	DXP vs Control/IRI	p=0.256	Rank-biserial r	-0.378	[-0.731, 0.135]
**Oedema**	PFD+DXP vs Control/IRI	p<0.001	Rank-biserial r	-1.000	[-1.000, -1.000]
**Oedema**	PTX+DXP vs Control/IRI	p=0.020	Rank-biserial r	-0.689	[-0.881, -0.303]
**Tunica albuginea separation/haemorrhage**	Sham vs Control/IRI	p<0.001	—	—	—
**Tunica albuginea separation/haemorrhage**	PFD vs Control/IRI	p=0.043	Rank-biserial r	-0.611	[-0.847, -0.175]
**Tunica albuginea separation/haemorrhage**	PTX vs Control/IRI	p=0.143	Rank-biserial r	-0.481	[-0.791, 0.023]
**Tunica albuginea separation/haemorrhage**	DXP vs Control/IRI	p=1.000	Rank-biserial r	0.300	[-0.220, 0.687]
**Tunica albuginea separation/haemorrhage**	PFD+DXP vs Control/IRI	p=0.008	Rank-biserial r	-0.767	[-0.913, -0.445]

Histopathological parameter	Comparison	p value	Effect-size metric	Estimate	95% CI
**Tunica albuginea separation/haemorrhage**	PTX+DXP vs Control/IRI	p=1.000	Rank-biserial r	-0.156	[-0.598, 0.360]
**Total pathology score**	Sham vs Control/IRI	p<0.001	—	—	—
**Total pathology score**	PFD vs Control/IRI	p=0.002	Rank-biserial r	-0.944	[-0.981, -0.847]
**Total pathology score**	PTX vs Control/IRI	p=0.012	Rank-biserial r	-0.840	[-0.943, -0.586]
**Total pathology score**	DXP vs Control/IRI	p=1.000	Rank-biserial r	-0.244	[-0.654, 0.277]
**Total pathology score**	PFD+DXP vs Control/IRI	p=0.001	Rank-biserial r	-0.989	[-0.996, -0.968]
**Total pathology score**	PTX+DXP vs Control/IRI	p=0.002	Rank-biserial r	-0.956	[-0.984, -0.876]

For each histopathological outcome, the five prespecified Control/IRI-versus-treatment Mann–Whitney U comparisons were Bonferroni-adjusted within the outcome (adjusted p=min[5×raw p, 1]). Two-sided asymptotic p values without continuity correction were used. The Sham-versus-Control/IRI comparison was analysed separately as a model-induction contrast.

Rank-biserial correlations were estimated from average ranks. Negative values indicate lower scores in the treatment group than in the Control/IRI group. The 95% confidence intervals were estimated using the normal approximation via Fisher transformation and were not multiplicity-adjusted.

Abbreviations: CI, confidence interval; DXP, dexpanthenol; IRI, ischaemia–reperfusion injury; PFD, pirfenidone; PTX, pentoxifylline.

Supplementary Table S4. Direct combination-versus-component comparisons for biochemical and histopathological outcomes

Parameter	PFD+DXP vs PFD	PFD+DXP vs DXP	PTX+DXP vs PTX	PTX+DXP vs DXP
**IL-1β**	p=0.021	p=0.315	p=0.833	p=0.345
**IL-10**	p=0.705	p=0.575	p=0.942	p=0.965
**MDA**	p=0.998	p<0.001	p=0.527	p=0.767
**eNOS**	p=0.137	p=0.385	p=0.110	p=1.000
**Vasocongestion**	p=0.717	p=0.239	p=0.793	p=1.000
**Inflammation**	p=1.000	p=0.961	p<0.001	p=0.009
**Desquamation**	p=1.000	p=0.002	p=1.000	p=0.002
**Oedema**	p=0.020	p=0.002	p=1.000	p=0.301
**Tunica albuginea separation/haemorrhage**	p=1.000	p=0.007	p=1.000	p=0.615
**Total pathology score**	p=0.449	p<0.001	p=0.701	p<0.001

Multiplicity-adjusted p values are reported. For IL-1β, Games–Howell post-hoc p values are shown; for IL-10 and MDA, Tukey HSD p values are shown. For eNOS and the histopathological outcomes, two-sided asymptotic Mann–Whitney U p values without continuity correction were Bonferroni-adjusted across the four direct combination-versus-component contrasts within each outcome (adjusted p=min[4×raw p, 1]). Statistical significance was predefined as p<0.050.

Abbreviations: DXP, dexpanthenol; eNOS, endothelial nitric oxide synthase; IL, interleukin; MDA, malondialdehyde; PFD, pirfenidone; PTX, pentoxifylline.
